# Evidence for the transfer of methadone and EDDP by sweat to children’s hair

**DOI:** 10.1007/s00414-021-02576-1

**Published:** 2021-04-05

**Authors:** Katharina Feld, Patrick Dahm, Tobias Kieliba, Axel Klee, Markus A. Rothschild, Hilke Andresen-Streichert, Justus Beike

**Affiliations:** 1 Institute of Legal Medicine, Medical Faculty, University of Cologne, Melatengürtel 60/62, 50823 Cologne, Germany; 2 Department of Dependency Diseases and Psychotherapy, LVR-Clinic Bonn, Bonn, Germany; 3 Institute of Legal Medicine, University Hospital Münster, Münster, Germany

**Keywords:** Sweat patches, Methadone to EDDP ratio, Children, Hair

## Abstract

In cases where there is a question as to whether children have come into contact with drugs, examinations of their scalp hair are frequently carried out. Positive test results are often discussed in the forensic community due to the various possible modes via which drugs and their metabolites can be incorporated into the hair. These include drug uptake by the child (e.g. oral ingestion or inhalation), but also contamination of hair via contact with the sweat from drug users. In this study, the possibility of methadone and its metabolite EDDP being incorporated into children’s hair by contact with sweat from persons undergoing opiate maintenance therapy (methadone) was examined. The transfer of methadone and EDDP via sweat from methadone patients (*n* = 15) to children’s hair was simulated by close skin contact of drug-free children’s hair, encased in mesh-pouches, for 5 days. Sweat-collecting patches (hereafter referred to as ‘sweat patches’) were applied to the test persons’ skin. One strand of hair and one sweat patch were collected daily from each patient. Analyses were performed using GC–MS/MS (hair) and LC–MS/MS (serum, sweat patches). After 4 days of skin contact, methadone was detectable in the formerly drug-free hair strands in all 15 study participants. EDDP was detectable in 34 of 75 hair strands, with the maximum number of positive results (11 EDDP-positive hair strands) being detected after 5 days. These results show that transfer of methadone and EDDP to drug-free hair is possible through close skin contact with individuals taking part in methadone substitution programmes. A correlation between serum concentration, sweat concentration and substance concentration in hair strands could not be demonstrated, but a tendency towards higher concentrations due to longer contact time is clearly evident.

## Introduction

Cases concerning the detection of drugs and metabolites in children´s hair are frequently discussed within the forensic and toxicological science community, as well as receiving attention from the media and political arenas [1, 2]. In 2011, the Institute of Legal Medicine at the University Hospital of Cologne has offered a routine service providing a test protocol for hair samples from children from families with known drug abuse or whose family members participate in a drug substitution therapy. In the course of these measurements, various drugs of abuse were detected in the children’s hair. The results of such hair analyses are often used by youth welfare offices to assess the risk to the child’s welfare [3, 4]. There is still, however, an uncertainty in the interpretation of the results, particularly in cases where additional metabolites were detected in a child’s hair [5]. Of particular interest is whether the children are deliberately or accidentally dosed with methadone or other drugs, for instance by a parent or guardian, as would be suggested by the presence of metabolites in the samples. Alternative scenarios are ingestion via accidental intake or external contamination of the child’s hair [3, 6]. Certainly, drug transfer during pregnancy and via breastfeeding has to be taken into consideration [7, 8].

Excessive sweating, known to be a side effect of methadone therapy, has resulted in both methadone and EDDP (2-ethylidene-1,5-dimethyl-3,3-diphenylpyrrolidine) being detected in the sweat of substitution patients [9]. Under such conditions, the transfer of these substances via sweat to the child’s hair can be regarded as plausible [3, 7]. In cases where parents are found to be responsible of potential neglect of a child’s wellbeing, there may be far-reaching consequences for the family, such as the child being removed into the custody of child protective services.

According to Kintz et al. sweat contamination could be considered one reason resulting in positive hair tests in children [10]. Nevertheless, incorporation of methadone and EDDP into a child’s hair had not as yet been proven [3], or had thus far been excluded [11]. The aim of this study was to examine whether the transfer of methadone and its metabolite EDDP is possible into a child’s hair from the sweat of methadone maintenance patients, and if the concentrations or ratios in hair due to sweat contamination would be different to the concentrations or ratios resulting from the ingestion of methadone. Such results could help avoid erroneous decisions, in terms of child welfare, due to incorrect or over-estimated interpretation of hair analysis results.

## Material and methods

### Clinical part

#### Study design

The transfer, via sweat from substitution patients to a child’s hair, of racemic methadone (d,l-methadone) or levomethadone (l-methadone) and their shared metabolite EDDP, was simulated by means of test subjects being exposed to prolonged dermal contact with drug-free children’s head hair. The children’s head hair, donated by the Bundesverband für Zweithaar-Spezialisten e.V., when the hair length was insufficient for the production of wigs, received ethical committee approval for its use in the study.

**Fig. 1:**
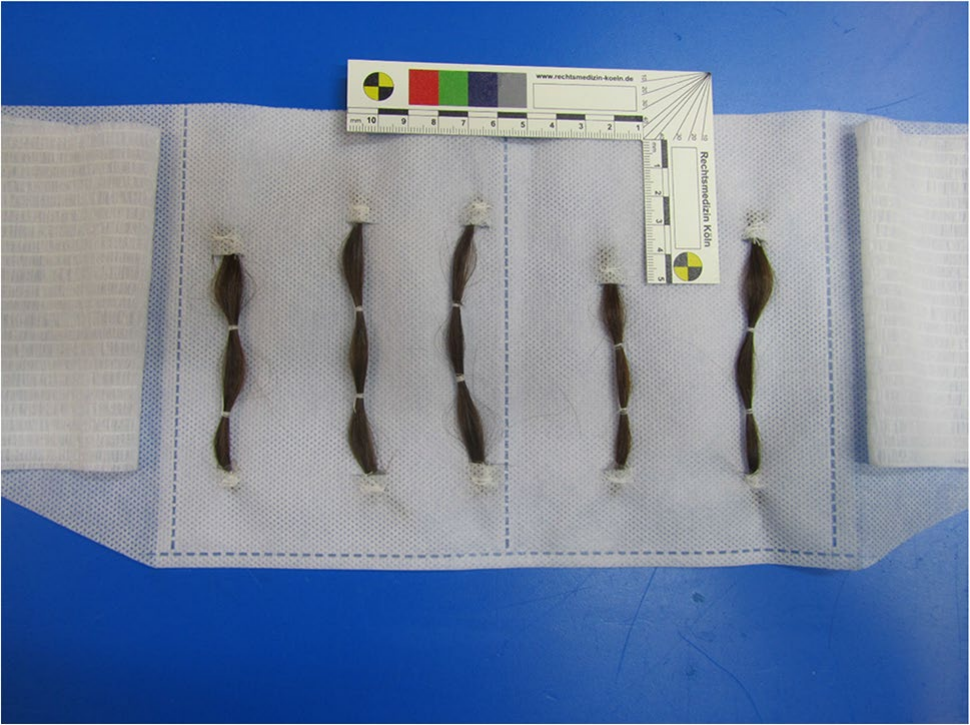
Pouch with 5 inserted hair strands

Hair/skin contact was achieved via mesh-encased belted pouches containing five strands (average weight of 0.7 g per strand) of drug-free children’s head hair (checked in advance by GC–MS/MS for the absence of methadone and EDDP) (Fig. 1). The belt was worn around the abdomen, enabling direct contact between the hair and the lower torso (abdomen, back or flanks). The original study design intended positioning the mesh pouches against the participant’s back. However, as the belt was prone to slip around the torso, positioning the pouches around the abdominal area gave the best and consistent positioning to maintain skin/hair contact throughout the study period. This hair-belt was worn for 6 days and was only removed for the purposes of bathing or showering.

Additional sweat-absorbing patches (the so-called sweat patches) were placed on the test subject’s skin (on their back) in order to collect the subject’s sweat in a ‘pure’ form [12].

**Fig. 2:**
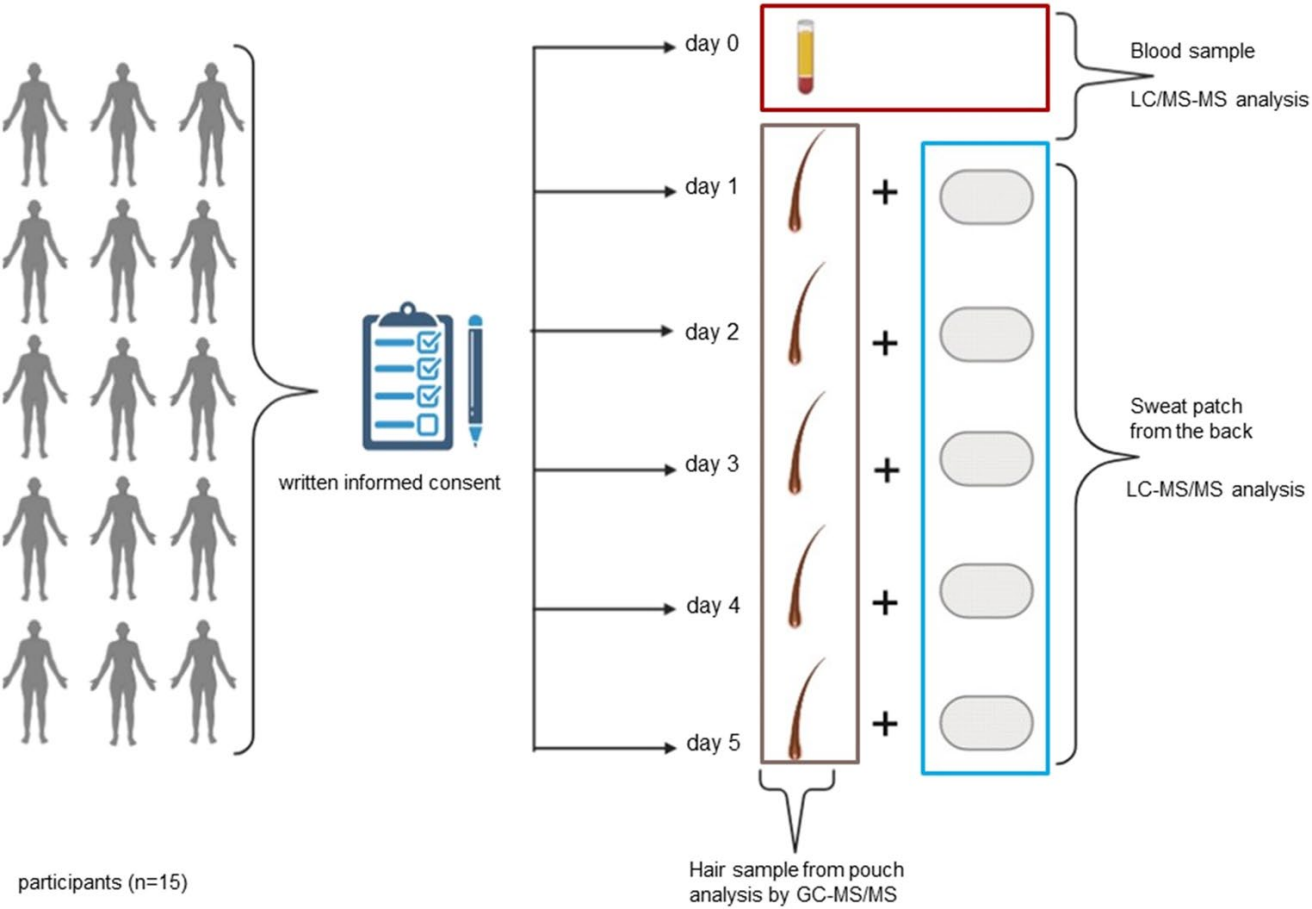
Study protocol (created in BioRender.com)

A blood sample was collected from each participant at the beginning of the study prior to ingesting their daily dose of d,l-methadone/l-methadone. The sample was analysed with regard to methadone and EDDP blood concentrations. Hair samples were not taken from the participants as no consent had been obtained for hair sampling (for an overview of the study design see Fig. 2).

#### Study group

Following ethical approval from the University of Cologne Medical Faculty ethical committee, 15 test persons were selected from a group of patients who take d,l-methadone or l-methadone in a medically indicated and controlled manner within the framework of weaning from their opiate dependence. Selection criteria included the ability to give consent; over the age of majority; the ability to provide and understand the information used as a basis for inclusion; a stable adjustment to methadone as part of the substitution programme for opiate addicts; consent to voluntary blood sampling and the ability to adhere the study requirements.

The study was registered with the German Clinical Trials Register under (DRKS00009854).

The final study group consisted of 15 persons (10 male and 5 female); each of whom had been in a methadone substitution programme for between two to 15 years. Eleven subjects received l-methadone solution (daily oral dose between 6 and 95 mg l-methadone) and 4 subjects received d,l-methadone as Methaddict® HEXAL tablets (daily oral dose 32.5 mg to 140 mg d,l-methadone). For an overview of the study group, see Table 1.

**Table 1:** Study group, dosage of substitution and methadone/EDDP in serum

Individual no	Sex	Age (years)	BMI (kg/m^2^)	Medication	Daily oral dose (mg/kg BW)	Methadone in serum (µg/L)	EDDP in serum (µg/L)	Ratio methadone/EDDP
1	m	50	38	l-methadone	0.58	177.6	18.7*	9.50
2	m	50	32	l-methadone	0.18	172.1	10.4*	16.55
3	m	54	23	l-methadone	0.26	101.5	10.2*	9.95
4	f	35	18	d,l-methadone	0.76	87.8	10.0*	8.78
5	m	46	28	l-methadone	0.62	291.1	18.8*	15.48
6	m	48	30	l-methadone	0.42	305.0	18.9*	16.14
7	f	26	43	d,l-methadone	1.14	633.5	47.2	13.42
8	f	57	29	d,l-methadone	2.06	464.1	28.3	16.40
9	m	57	23	l-methadone	0.49	389.7	18.8	20.73
10	m	61	28	l-methadone	0.26	268.3	8.5*	31.56
11	m	41	31	l-methadone	0.38	374.9	15.1*	24.83
12	f	40	27	l-methadone	1.27	527.9	47.0	11.23
13	m	45	23	l-methadone	0.63	155.1	15.0*	10.34
14	m	51	23	d,l-methadone	0.60	274.8	13.2*	20.82
15	f	49	23	l-methadone	0.10	15.6*	n.d	9.50

*m* male, *f* female, *BMI* body mass index, *BW* body weight, *value below LOQ, *n.d.* not detected (below LOD); (LOD = 6.8 and 6.3 µg/L for methadone and EDDP, respectively, LOQ = 25 µg/L for both analytes)

#### Sample collection

Hair strand from hair-pouch: one hair strand each day, from day 2 to day 6 of the study period.

Sweat patch: one sweat patch each day, from day 2 to day 6 of the study period.

Blood sample (serum tubes): one sample on day 1, prior to consuming the daily dose of d,l-methadone or l-methadone, respectively.

### Analytical procedure

The blood specimen (serum), drug-free hair and exposed hair from hair pouches were prepared for toxicological analysis and tested for the presence and concentration of methadone and EDDP by validated and court-approved chromatographic analysis procedures (according to DIN EN ISO/IEC 17,025). For the determination of methadone and EDDP in sweat (patches), a method was established and validated, based on the method published by Brunet et al. 2008 [13]. An enantioselective differentiation of l-methadone and d-methadone was not performed.

#### Chemicals, reagents and materials

Certified reference standards of d,l-methadone and EDDP (each 1 mg/mL in methanol) as well as deuterated standards (d,l-methadone-d9, EDDP-d3; each 0.1 mg/mL in methanol) were purchased from Lipomed AG (Arlesheim, Switzerland). For calibration purposes, standard solution mixtures and internal standard solutions (ISTD, containing the deuterated analogues) were prepared in methanol.

Methanol (MeOH, HPLC gradient grade), 2-propanol (HPLC grade), dichloromethane (DCM, HPLC grade), water (Ultra LC–MS grade), acetonitrile (Ultra LC–MS grade) and lactic acid 80% were supplied by Carl Roth (Karlsruhe, Germany). Ethyl acetate (CHROMASOLV Plus HPLC 99.9%) was purchased from Honeywell (Seelze, Germany), and formic acid (LC–MS grade) from Thermo Scientific (Rockford, IL, USA).

All other chemicals were of analytical grade: ammonium hydroxide solution (25%), 0.1 M hydrochloric acid (titration solution) and acetic acid (100%) provided by Carl Roth; sodium hydroxide (pure pellets), sodium chloride, ammonium chloride, sodium acetate, potassium dihydrogen phosphate (KH_2_PO_4_) and sodium dihydrogen phosphate monohydrate (NaH_2_PO_4_*H_2_O) provided by Merck (Darmstadt, Germany). Urea (99.5%) was purchased from GE Healthcare Bio-Science AB (Uppsala, Sweden).

0.1 M and 1 M acetic acid solutions (HAc), as well as 1 M and 2 M NaOH solutions, were prepared in bi-distilled water. 0.1 M phosphate buffer was made up of NaH_2_PO_4_*H_2_O in bi-distilled water and the pH was adjusted to 6.0 by adding 1 M NaOH (utilising a pH electrode). 0.5 M sodium acetate buffer (pH 4.0) was made up of 0.089 mol sodium acetate and 0.411 mol acetic acid in bi-distilled water and the pH was adjusted to 4.0 by adding 1 M acetic acid solution (utilising a pH electrode, Knick; Berlin). Artificial sweat solution was prepared according to Brunet et al. (2008) [13]. High-purity water (generated by a GenPure xCAD system; Thermo Fisher Scientific, Dreieich, Germany) was used for solid-phase extraction (SPE) washing purposes.

Sweat patches (PharmCheck Sweat Patch Kit) were purchased from PharmChem Inc. (Fort Worth, TX, USA).

Drug-free children’s scalp hair was provided by an organisation that collects hair donations from children to make wigs for children who have lost their hair as a result of cancer treatment (Bundesverband für Zweithaar-Spezialisten e.V.). The hair, provided from four donors, was of insufficient length to manufacture wigs. This hair was analysed for the absence of drugs of abuse prior to it being used in this study. Strands of one pouch each came from a single source donation.

#### Validation

Validations were conducted according to guidelines of the Society of Toxicological and Forensic Chemistry (GTFCh) [14, 15]. In brief, they covered linearity, limit of detection (LOD), limit of quantification (LOQ), accuracy with respect to precision and trueness, as well as recovery, stability and matrix effects (serum, LC–MS/MS). Sensitivity was evaluated by determining the limits of detection (LOD) and quantification (LOQ) for methadone and EDDP. For sweat patches and hair samples, the lowest concentration with acceptable peak shape, chromatographic resolution, retention time, qualifier transition ratios and a signal to noise of at least 3:1 was defined as LOD. LOQ was defined with signal to noise of at least 9:1 for sweat patches. For hair samples, it was defined as the concentration of a quality control (QC), which could be measured with sufficient bias after fivefold measurement. LOD and LOQ for serum samples were established according to DIN32645.

Testing for linearity was done by analysing the calibration in 2 (sweat patches), 5 (hair samples) or 6 (serum samples) replicates, followed by evaluating linearity by the Mandel test, outliers by the Grubbs test and variance homogeneity by the Cochran test (each 99% level of significance). Accuracy and imprecision were evaluated over the linear range using two QC samples at higher and lower concentrations of d,l-methadone and EDDP. QC samples were measured on 3 (patches), 5 (hair) or 8 (serum) samples on different days in duplicates. The determination of the concentrations was based on a daily calibration.

Additionally, a possible generation of EDDP formation from methadone by hot GC inlet, as described elsewhere for urine testing, was evaluated [16]. At the chosen, lowered inlet temperature (200 °C), no significant influence was observed on the test results.

All statistical evaluations were undertaken using the Valistat software version 2.04 provided by Arvecon (Walldorf, Germany), or Microsoft® Excel 2016.

#### Blood analysis

##### Sample preparation

Quantification in serum was performed by a validated routine method used to determine several benzodiazepines, z-drugs, d,l-methadone and their metabolites in a target screening by LC–MS/MS combined with a MultiPurposeSampler (MPS). For this study, only d,l-methadone and EDDP in serum were analysed. Briefly, after centrifugation, 0.25 mL serum supernatant were separated, to which 1 mL 0.1 M phosphate buffer (pH 6.0) and 25 µL of the internal standard mixture of d,l-methadon-d9 and EDDP-d3 (2 mg/L in acetonitrile, final concentration 50 µg/L) were added. Further sample preparation was done fully automatically by the MPS device. Conditioning of SPE cartridges (Chromabond HR-XC, 3 mL /60 mg, 45 µm; Macherey–Nagel, Düren, Germany) was by MeOH, high-purity water and 0.1 M phosphate buffer. After sample loading, cartridges were washed with phosphate buffer, MeOH/water (30/70, v/v), 0.1 M hydrochloric acid, an additional time with MeOH/water (30/70) and finally with 0.1 mL MeOH followed by drying with nitrogen. Analytes were eluted with ethyl acetate/ammonium hydroxide 25% (100/4, v/v). After evaporation, the residue was dissolved with water (Ultra LC–MS grade) and acetonitrile (85/15, v/v). Five microlitres were injected into the LC–MS/MS system.

##### Instrumentation

Analysis of serum extracts was done using an HPLC system (model 1200SL) and a tandem mass spectrometer (model 6460A) from Agilent Technologies (Waldbronn, Germany), equipped with electrospray ionisation (ESI) source and integrated jet stream technology. MassHunter Workstation Software LC/QQQ Data Acquisition (version B.08.02, Agilent Technologies) was used to control the system. SPE was conducted fully automatically using a MPS Autosampler controlled by the Maestro Software (version 1.4.55.1, all Gerstel, Mühlheim/Ruhr, Germany).

##### Quantification

Chromatographic separation of serum extracts was performed with a Nucleoshell RP 18 EC (150 mm × 2.0 mm, 2.7 µm; Macherey–Nagel) column (flow rate 0.3 mL/min; 30 °C oven temperature). As mobile phase, water (Ultra LC–MS grade) with 0.1% formic acid (A) and acetonitrile with 0.1% formic acid (B) was used. Initial conditions were 10% of solvent B, increased over 7 min to 50%, and further increased over 4 min to 90%, where it was held for 2 min. The system was returned to initial conditions and held for a further 2 min, resulting in a total runtime of 20 min.

ESI was operated in positive mode and parameters were set to 3500 V capillary voltage, 325 °C gas temperature, 12 L/min sheath gas flow, 45 psi nebulizer, 375 °C sheath gas temperature, 10 L/min gas flow and 500 V nozzle voltage. Nitrogen was used as collision gas, applying different collision energies (CE). Quantifier transitions were 310.2 > 265.1 (CE 9 V) for methadone, 319.3 ≥ 268.2 (CE 9 V) methadone-d9 as well as 278.2 ≥ 249.1 (CE 21 V) for EDDP, 282.2 ≥ 235.2 (CE 29 V) EDDP-d3. Qualifier transitions were 310.2 ≥ 105 (CE 25 V) for methadone, as well as 278.2 ≥ 234.1 (CE 29 V) for EDDP.

Quantification of d,l-methadone and EDDP in serum samples was implemented with a calibration range from 25 to 500 µg/L. During validation, a LOD of 6.8 and 6.3 µg/L for d,l-methadone and EDDP, respectively, as well as a LOQ of 25 µg/L for the detection of both analytes, was determined.

The external QC samples (OSD level 2 and level 4) were purchased from Medichem (Steinenbronn, Germany).

#### Sweat patch analysis

##### Sample preparation

Sweat patches were folded and placed in a test tube to which sodium acetate buffer (pH 4.0) was added. Sealed tubes were shaken on a horizontal shaker for 10 min. Following this, the buffer was transferred into a further test tube, the extraction procedure with buffer was repeated and the aliquots were combined. Conditioning of SPE cartridges (Strata-X-Drug B, 3 mL / 60 mg, 33 µm; Phenomenex, Aschaffenburg, Germany) was by MeOH and sodium acetate buffer. After sample loading, cartridges were washed with sodium acetate buffer, MeOH/water (30/70, v/v), 0.1 M hydrochloric acid, an additional time with MeOH/water (30/70), then 0.1 mL MeOH followed by drying in nitrogen. Analytes were eluted using ethyl acetate/25% ammonium hydroxide (98/2, v/v). Eluates were evaporated to dryness with nitrogen at room temperature. Dried residues were dissolved in water (Ultra LC–MS grade) and acetonitrile (85/15, v/v). Five microlitres were injected into the LC–MS/MS system.

##### Instrumentation

The LC–MS/MS system and software were identical to that used in the analysis of serum (see the ‘Blood analysis’ and the ‘Instrumentation’ sections). SPE was manually conducted using a VacMasertTM 10 vacuum manifold (Biotage, Uppsala, Sweden) to clean up and concentrate d,l-methadone and EDDP from sweat patches.

##### Quantification

Chromatographic separation of sweat patch extracts was performed with a Kinetex® Polar C 18 column (100 mm × 2.1 mm, 2.6 µm; Phenomenex). The chromatographic conditions, as well as ionisation settings and transitions, were identical to the quantification of serum (see the ‘Blood analysis’ and the ‘Quantification’ sections). Limits of detection were 2.5 ng/patch and 1.5 ng/patch; limits of quantification were 5 ng/patch and 2.5 ng/patch for d,l-methadone and EDDP, respectively.

Stock solutions of d,l-methadone and EDDP were combined and diluted with methanol to yield working solutions (0.05 to 10.0 µg/mL for d,l-methadone and 0.025 to 5.0 µg/mL for EDDP). Blank sweat patches were evenly moistened with 750 µL of artificial sweat and allowed to dry for 2 h at room temperature.

One hundred microlitres of QC solutions, working solution and internal standard solution were added to sweat patches. QC solutions were separately diluted, daily prepared and added to blank sweat patches to yield a total of 15 ng, 400 ng for d,l-methadone and 7.5 ng, 200 ng for EDDP.

Deuterated standards were diluted to a concentration of 20 ng/patch and 10 ng/patch for d,l-methadone-d9 and EDDP-d3 respectively.

During validation, calibration curves were freshly prepared by spiking sweat patches or elution solvent (ethyl acetate/25% ammonium hydroxide); sweat patches were premoistened with artificial sweat and with a working solution to obtain 5 to 1,000 ng/patch for d,l-methadone and 2.5 to 500 ng/patch for EDDP. The relative response of matrix calibration and solvent calibration was found to be comparable. Subsequently, solvent calibration curves were used as the number of sweat patches was restricted and the usage of solvent calibration was more practical.

#### Hair analysis

##### Sample preparation

One half of each hair strand was used; the other part was retained for any necessary reanalyses. The first aliquot used the whole length of strand, washed two times with 5 mL DCM each and dried room temperature (approx. 20 min). Subsequent grinding was performed up to 5 min in a ball mill (MM 2000 from Retsch, Haan, Germany) with stainless steel balls. Twenty-five milligrammes of the powdered hair were transferred to a test tube, fortified with 25 µL ISTD solution (resulting in a concentration of 500 pg/mg d,l-methadon-d9 and EDDP-d3). For hair extraction, 1 mL of 0.1 M HCl was added and closed tubes were placed in an ultrasonic bath for 3 h. After centrifugation, supernatants were neutralised by adding 1 M NaOH and adjusted to pH 6.0 with phosphate buffer.

Clean-up of the prepared hair samples was done by SPE with Strata Screen-C cartridges (3 mL /200 mg, 55 μm; Phenomenex, Aschaffenburg, Germany) following an in-house protocol. In brief, sorbent was conditioned with MeOH and 0.1 M phosphate buffer; after the sample load, it was washed with 0.1 M HAc, high-purity water and MeOH, dried and eluted with alkaline elution mixture (DCM, 2-propanol and 25% ammonium hydroxide (80/20/2, v/v/v)). The extracts were then evaporated to dryness with nitrogen at 30 °C and reconstituted in 25 µL ethyl acetate. Prepared sample extracts were carefully crimped into amber glass vials.

##### Instrumentation

Sample SPE clean-up of hair samples was performed in an automated ASPEC GX 274 System with two 406 Dual Syringe Pumps (Gilson, Limburg-Offheim, Germany).

Measurements of hair sample extracts were conducted on a 7890A / 7000B gas chromatography-tandem mass spectrometry (GC–MS/MS) system equipped with a Split/Splitless inlet (Agilent Technologies, Waldbronn, Germany). A deactivated 4.0 mm ID Single Taper Liner with CarboFrit (Restek, Bad Homburg, Germany) and a GC column Zebron ZB-5MSi 15 m × 0.25 mm × 0.25 µm (Phenomenex) were used. The MassHunter Workstation Software GC/QQQ Data Acquisition (version B.05.02, Agilent Technologies) was used to control the GC–MS/MS-system.

##### Quantification

One microliter of the reconstituted extract was injected splitlessly at 200 °C for transfer onto the GC column. Separation was performed with a constant helium flow of 1.1 mL/min and the following temperature program: 80 °C (1 min), 15 °C/min, 290 °C (3 min), resulting in a total GC run time of 18 min. Nitrogen was used as the collision gas. Quantifier transitions were 72 ≥ 56 (CE 20 V) for methadone, 78 ≥ 59 (CE 20 V) methadone-d9 as well as 277 ≥ 220 (CE 20 V) for EDDP, 280 ≥ 220 (CE 20 V) EDDP-d3. Qualifier transitions were 294 ≥ 223 (CE 10 V) for methadone, 303 ≥ 226 (CE 10 V) methadone-d9 as well as 262 ≥ 170 (CE 20 V) for EDDP, 265 ≥ 171 (CE 20 V) EDDP-d3.

Quantification of d,l-methadone and EDDP in hair samples was implemented within a calibration range of 50 to 5000 pg/mg. Pooled blank hair fortified with calibration standards and ISTD was used for calibration. Matrix blank samples that had been tested as negative were taken from volunteers of the Institute of Legal Medicine (Cologne, Germany). Each sample sequence contained a blank hair sample (including ISTD) as well as external and internal QC samples. The external QC sample (DHF 1/18-B) was purchased from ACQ SCIENCE (Rottenburg-Hailfingen, Germany). An internal QC sample, consisting of pooled drug-positive hair (containing 586 pg/mg d,l-methadone and 689 pg/mg EDDP), was prepared by the Institute of Legal Medicine (Cologne, Germany). During validation, a LOQ of 50 pg/mg and a LOD of 25 pg/mg for the detection of d,l-methadone and EDDP were determined. Accuracy of quantitation above an upper limit of detection, by means of linear extrapolation, was demonstrated for 10,000 and 15,000 pg/mg (96.5–99.9%).

#### Merging of data

The results of the analyses of hair and sweat patches were recorded and are published here in anonymous or pseudonymised form and have been compared with data obtained from test subjects’ blood samples and information about body weight and daily dose of d,l-methadone/l-methadone.

## Results

All 15 persons completed the 6-day study phase. Beginning at the second study day (day 1), one strand of hair from the hair pouch and one sweat patch were collected each day (Fig. 2), in order to detect the variation in concentration of substances with respect to exposure time. Thus, in total, we received one blood sample, 5 sweat patches and 5 hair strands from the hair pouch from each participant. The characterisation of the study group, and the daily methadone dose and the results of the serum tests are summarised in Table 1.

### Methadone and EDDP in the blood

The concentrations of methadone were in the range of 15.6 µg/L to 633.5 µg/L (mean = 282.6 µg/L). The dosage of methadone ranges from 0.1 to 2.06 mg/kg of body weight. There was no clear correlation between methadone dosage and the concentrations of methadone and EDDP in the blood. The participant with the lowest dose of 0.1 mg l-methadone/kg had the lowest blood concentrations of methadone and EDDP. However, the person with the highest dose of 2.06 mg methadone/kg did not have the highest blood concentrations (*R*^2^ = 0.4194). Moreover, persons with the same dose (no. 3 and no. 10; no. 5 and no. 13) showed clearly different blood concentrations.

### Methadone and EDDP in sweat patches

Both methadone and EDDP were detectable in all sweat patches collected from all study participants (Table 2). A clear correlation between methadone dosage and the amounts of methadone and EDDP in the sweat patches could not be observed. Only in the participant with the lowest dosage (no. 15) were the lowest amounts of methadone and EDDP found in the sweat patches.

**Table 2:** Methadone and EDDP in sweat patches

	Patch 1 (ng/patch)		Patch 2 (ng/patch)		Patch 3 (ng/patch)		Patch 4 (ng/patch)		Patch 5 (ng/patch)	
Individual no	Methadone	EDDP	Methadone	EDDP	Methadone	EDDP	Methadone	EDDP	Methadone	EDDP
1	523	4.5	961	7.5	1314**	11	299	5.4	2116**	15
2	193	2.9	238	3.2	724	5.3	824	6.5	976	6.6
3	30	1.9*	72	2.1*	62	2.1*	92	2.3*	223	2.7
4	205	2.7	348	3.3	825	5.8	574	4.4	676	4.8
5	131	2.3*	170	2.8	400	3.4	342	3.3	302	3.3
6	187	2.5	294	2.8	438	3.6	468	3.7	362	3.4
7	727	6.7	2005**	15	4780**	31	3676**	20	4593**	26
8	34	2.2*	135	3.2	245	4.0	348	5.1	822	9.5
9	147	2.2*	526	3.6	868	4.7	919	4.7	1102**	4.8
10	1622**	5.8	2541**	7.1	1213**	4.9	4021**	10	4389**	10
11	339	3.2	550	4.3	706	4.9	1221**	6.5	1321**	5.7
12	211	3.1	898	8.1	1759**	13	1383**	9.6	1466**	9.5
13	562	4.6	874	6.5	1424**	10	1927**	13	2356**	14
14	542	3.4	896	4.8	1231**	5.9	1351**	5.8	1705**	6.7
15	19	1.8*	31	1.9*	47	1.9*	116	2.1*	88	2.0*

^*^Value below LOQ (5.0 ng/patch for methadone and 2.5 ng/patch for EDDP) and above LOD (2.5 ng/patch for methadone and 1.5 ng/patch for EDDP)

^**^Approximated value, concentration above the highest calibration point (1000 ng/patch for methadone and 500 ng/patch for EDDP)

The highest amounts of methadone and EDDP in the sweat patches were found in subject no. 7, who also had the highest body mass index (BMI). However, no other influence could be found between BMI and the amount of methadone and EDDP per patch.

**Fig. 3:**
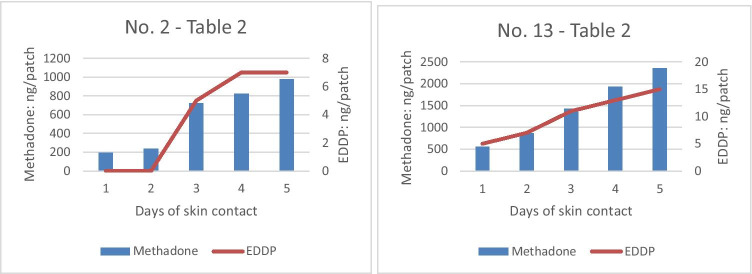
Exemplary courses of methadone (blue bars, left axis) and EDDP (red line, right axis) concentrations in sweat patches over the study period in two patients

In general, an increase in the amount of methadone per sweat patch from day 2 to 6 could be observed when looking at the data from individual participants. However, in 7 persons, there are one or two outliers each, which do not strictly follow this trend. In 10 subjects, the highest amount of methadone per sweat patch was present on day 6, as expected. In 4 subjects, the highest dosage was already observed on day 4 and in one subject on day 5. Exemplary data of methadone and EDDP concentrations in sweat patches over the study period can be seen in Fig. 3.

The amounts of EDDP per sweat patch were clearly lower than the amounts of methadone. They varied less than the methadone amounts and did not follow a clear trend. The highest levels of EDDP per sweat patch were found in only 6 people on the last day of the study. The median ratios of methadone to EDDP per sweat patch for all persons and days ranged from 15 to 402. However, within one person, the ratios of methadone to EDDP per sweat patch varied less (Table 3). One could distinguish three groups here: (a) methadone to EDDP ratio mean < 100 (4 persons), (b) methadone to EDDP ratio median > 100 to < 200 (9 persons) and (c) methadone to EDDP median > 200 (2 persons). A correlation between the ratio of methadone to EDDP per sweat patch and d,l-methadone or l-methadone medication cannot be deduced. There was also no correlation between the ratio of methadone to EDDP per sweat patch and the methadone dosage or the BMI or the sex of the test persons.

**Table 3:** Ratios of methadone and EDDP in sweat patches

Individual no	Ratio (methadone/EDDP)							
	Day 1	Day 2	Day 3	Day 4	Day 5	Minimum	Maximum	Mean
1	105	120	119	60	132	60	132	107
2	64	79	145	118	139	64	145	109
3	15	36	31	46	74	15	74	40
4	68	116	138	144	169	68	169	127
5	66	57	133	114	101	57	133	94
6	62	98	110	117	121	62	121	101
7	104	134	154	175	170	104	175	147
8	17	45	61	70	82	17	82	55
9	74	132	174	184	220	74	220	156
10	270	363	243	402	399	243	402	335
11	113	138	141	174	220	113	220	157
12	70	112	135	138	147	70	147	120
13	112	125	129	148	157	112	157	134
14	181	179	205	225	244	179	244	206
15	10	16	24	58	44	10	58	30

### Methadone and EDDP in hair samples after sweat contact

**Fig. 4:**
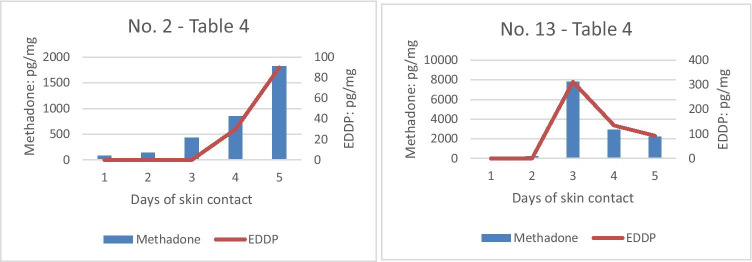
Exemplary courses of methadone (blue bars, left axis) and EDDP (red line, right axis) concentrations in hair over the study period in two patients

After 1 day of skin contact with the study participants, methadone was already detectable in 10 of 15 hair samples. After 4 days, methadone was detectable in the hair samples of all 15 study participants. Observation of EDDP provides a different picture: After 1 day of skin contact, EDDP could already be detected in 3 samples; the maximum of 11 EDDP-positive hair samples could be measured after 5 days of skin contact. In total, EDDP was detectable above the LOD in 34 of 75 hair samples (Table 4). Exemplary data on methadone and EDDP concentrations in hair samples over the study period can be seen in Fig. 4.

**Table 4:** Methadone and EDDP in hair samples

	Hair sample 1 (pg/mg)		Hair sample 2 (pg/mg)		Hair sample 3 (pg/mg)		Hair sample 4 (pg/mg)		Hair sample 5 (pg/mg)	
Individual no	Methadone	EDDP	Methadone	EDDP	Methadone	EDDP	Methadone	EDDP	Methadone	EDDP
1	475	25*	471	29*	2644	134	4298	156	1298	57
2	77	< LOD	140	< LOD	429	< LOD	854	30*	1825	90
3	< LOD	< LOD	< LOD	< LOD	< LOD	< LOD	46*	< LOD	29*	< LOD
4	164	< LOD	422	< LOD	415	< LOD	356	< LOD	275	< LOD
5	< LOD	< LOD	71	< LOD	182	< LOD	98	< LOD	55	< LOD
6	400	< LOD	1230	67	4418	143	3053	97	5229	178
7	1404	63	8285	307	9749	299	9104	276	11,880	398
8	< LOD	< LOD	162	26*	87	< LOD	322	< LOD	491	25*
9	38*	< LOD	240	< LOD	453	< LOD	491	< LOD	720	29*
10	583	< LOD	4959	165	2848	86	10,459	342	12,122	375
11	< LOD	< LOD	102	< LOD	361	< LOD	355	< LOD	835	36*
12	48	< LOD	269	< LOD	805	40*	962	30*	838	66
13	75	< LOD	243	< LOD	7801	312	2936	134	2212	92
14	1761	117	1069	43	953	< LOD	1004	40*	2107	76
15	< LOD	< LOD	25*	< LOD	84	< LOD	147	< LOD	157	< LOD

^*^Approximately, value below LOQ

LOD = 25 pg/mg; LOQ of 50 pg/mg (each methadone and EDDP)

As has already been demonstrated with the concentrations of methadone and EDDP in the blood samples and the amounts of methadone and EDDP in the sweat patches, there was no correlation between the dosage in mg/kg body weight and the concentrations of methadone and EDDP in the hair samples.

A connection between BMI and the methadone and EDDP concentrations in the hair has not been established either.

Looking at the data from individuals, trends of increasing methadone and EDDP concentrations over the 5 days were observed, with higher concentrations and clearer trends for methadone than for EDDP. With regard to methadone concentrations, there was a clear increasing trend in 4 persons (persons no. 2, 9, 11, 15). For the remaining sample series, there were several (between 1 and 3) concentrations that did not follow the rising trend.

The ratio of methadone to EDDP in hair could only be calculated in those samples where EDDP was also detectable. EDDP was not detectable in 41 of the 75 hair samples. Therefore, no methadone to EDDP ratio could be determined for 4 study participants (no. 3, 4, 5 and 15) (Table 5).

**Table 5:** Ratios of methadone and EDDP in hair samples

Individual no	Ratio (methadone/EDDP)							
	Day 1	Day 2	Day 3	Day 4	Day 5	Minimum	Maximum	Mean
1	**19**	**16**	**20**	**28**	**23**	**16**	**28**	21
2	n/a	n/a	n/a	**28**	**20**	**20**	**28**	24
3	n/a	n/a	n/a	n/a	n/a	n/a	n/a	n/a
4	n/a	n/a	n/a	n/a	n/a	n/a	n/a	n/a
5	n/a	n/a	n/a	n/a	n/a	n/a	n/a	n/a
6	n/a	**18**	**31**	**31**	**29**	**18**	**31**	27
7	**22**	**27**	**33**	**33**	**30**	**22**	**33**	29
8	n/a	**6**	n/a	n/a	**20**	**6**	**20**	13
9	n/a	n/a	n/a	n/a	**25**	n/a	n/a	n/a
10	n/a	**30**	**33**	**31**	**32**	**30**	**33**	31
11	n/a	n/a	n/a	n/a	**23**	n/a	n/a	n/a
12	n/a	n/a	**20**	**32**	**13**	**13**	**32**	21
13	n/a	n/a	**25**	**22**	**24**	**22**	**25**	23
14	**15**	**25**	n/a	**25**	**28**	**15**	**28**	23
15	n/a	n/a	n/a	n/a	n/a	n/a	n/a	n/a

In the EDDP-positive hair samples of the other 11 study participants, the ratios of methadone to EDDP were in a markedly narrower range compared to those in the sweat patches. The mean values varied between 13 and 31. A moderate correlation could be seen between hair and patch ratios in the 9 patients in which both were calculated (*R*^2^ = 0.4631).

Two of the 4 study participants, in whom EDDP was not detectable in any of the hair samples, showed low ratios (of < 100) of methadone to EDDP in the sweat patches. The other two of these 4 study participants had methadone to EDDP ratios between 100 and 200 in the sweat patches.

The present collective is too small for statistically sound statements. However, all results show a tendency and are indicative.

## Discussion

It has been suggested that drugs may be incorporated into hair: (i) from the blood during hair formation; (ii) from sweat and sebum after formation and (iii) from the external environment after formation and after the hair has emerged from the skin [17]. It must be noted also that hair from children is finer and more porous in comparison with that from adults, rendering the hair at higher risk of contamination by (own) sweat versus that of adults [8].

Our investigations have shown that the transfer of methadone and its metabolite EDDP to previously drug-negative hair is possible through close skin/sweat contact with persons treated with methadone. This finding is of great significance for the interpretation of the results of analyses on children’s hair. If the children have close physical contact with methadone substitution patients or methadone ingesting persons, e.g., with their parents, contamination of the child’s hair by methadone- and EDDP-containing sweat is clearly possible. The assumption that the detection of the metabolite EDDP in a child’s hair indicates that the child may have ingested methadone themselves, as has been previously expressed [11], is thus refuted. Previous authors have also stated that the detection of EDDP in addition to methadone is an indication of at least partial systemic intake of the drug [3, 18]. Results from this study, however, show that although the existence of EDDP proves methadone has been metabolised, it does not prove that the metabolism must have taken place in the body of the child in question.

A correlation between methadone dosage and the concentrations of methadone and EDDP found in the blood samples of study participants could not be established. This observation was also made in previous studies and can be explained by a high inter-individual variance in pharmacokinetic properties [19, 20]. However, the methadone and EDDP concentrations, as well as the methadone/metabolite ratios in serum, were in the same range as those published by previous authors (3.92–53.95 (through), 4.06–30.04 (peak) [21]; 5.6–15.1 [22]).

In the hair, the reported ranges of methadone concentrations were between 0.25 and 80.8 ng/mg; the ranges of EDDP between 0.05 and 7.76 ng/mg. The corresponding ratios in those studies (methadone-to-EDDP) were calculated as between 1.4 and 32.2 [9, 23–25]. Both ranges and ratios were in line with the results from our study (see Tables 4 and 5). Again, a correlation between methadone doses and hair concentrations could not be found, as previously stated [25, 26].

For the sweat patches, comparable data for methadone and EDDP are rare. Fucci et al. found 120 to 2160 ng methadone and 25–535 ng EDDP/patch in 10 patients [9]. Gambelunghe et al. determined 300–650 ng methadone and 50–90 ng EDDP/patch in 48 patients [27]. In these studies, the EDDP/methadone ratios were between 0.07 and 0.3 (corresponding to methadone/EDDP ratios of 14.3 – 3.3) [9, 27, 28]. In our study, methadone concentrations were in a broad, but comparable range (19–4389 ng/patch). However, the methadone/EDDP ratios were, with 15–402, in a considerably higher range, although the concentrations of EDDP in our patches were obviously lower than had previously been reported. Consequently, the methadone/EDDP ratios are higher and have to be interpreted with caution. The cause of this phenomenon was not clear. A methodological reason (analysis by LC–MS/MS vs GC/MS in the studies of [9] and [27]); higher ion suppression due to sweat/patch matrix [29] or instability of EDDP on the patch/on the skin have to be discussed. Additionally, in several patches, the concentration of EDDP lies below the LOQ and close to the LOD. It could be assumed that this had an impact on the ratios, even though the ratios of the individual patches were in fact inter-individually clearly different in our 15 patients, but intra-individually consistent. Moreover, the range of EDDP concentrations and methadone concentrations in sweat is in accordance (see Figs. 3 and 4). The differences in excretion (parent drugs are more likely to be encountered in sweat than polar hydrophilic metabolites [30]) had been previously discussed.

An additional aim of our study was to examine if the concentrations or ratios in hair due to sweat contamination would be different to the concentrations or ratios resulting from the ingestion of methadone. In the event of such a distinction being observed, a better interpretation of children’s hair results would be possible. As could be shown, there is a relevant overlapping of these levels, making discrimination between ingestion and contamination based on ratios impossible. Although this result is consistent, considering that a relevant part of methadone and EDDP in hair may be the result of sweat from the individual [17].

To examine the applicability of our data, results were compared to those from selected real cases of hair analysis in living children found in literature—although few cases were suitable for this kind of comparison—as most of the published results are related to fatal or non-fatal methadone intoxications in children [1, 7, 18]. In these scenarios, an additional contamination from body fluids or tissues would have to be considered, if the hair samples were obtained from children known to have had methadone in their body close to the time of hair sampling [6]. Additionally, in some cases, comparison was not possible due to concentrations of EDDP not having been determined/detected [2, 18, 31]. In their review article, Pragst et al. [3] provided comprehensive data for toxicological results of children’s hair. In 11 of 18 methadone positive children, EDDP was also measurable. Methadone concentrations of 0.156 to 2.16 ng/mg and EDDP concentrations of 0.011 to 0.074 ng/mg, respectively, led to EDDP/methadone-ratios of 0.010 to 0.12 (corresponding to methadone/EDDP-ratios of 100 to 8.07). These ratios are mainly in the range of those determined in this study for sweat patches (10 to 402)—with only one of 11 ratios below 10—and in the range of those ratios determined for sweat contaminated hair (6 to 33), with only two samples with ratios above 33 [3]. Therefore, in none of these data from hair samples published by Pragst et al. would the ratio be contradictory to an external contamination. Furthermore, Pragst et al. considered that, due to the higher concentrations of methadone in the children, Ch022, Ch059 and Ch040 with concentrations of 1.36, 0.38 and 0.29 ng/mg and without detection of EDDP, external contamination of the hair by methadone seems to be the main route of incorporation, as EDDP should be detectable in cases of dominating systemic uptake [3]. In our cohort, several sweat-contaminated hair samples (see Table 4) showed methadone concentrations above 0.29 ng/mg (i.e. 0.42 ng/mg (Ind. no. 2, sample 3); 0.35 ng/mg (Ind. no. 4, sample 4)), up to 58 ng/mg (Ind. no. 10, sample 1), where no EDDP was detectable. Based on our results, not only is an external contamination possible for these children by methadone itself, but also a contamination via sweat could be a possible explanation. Taking everything into account, we agree with these authors that a systemic uptake of methadone could not be proven.

Although thoroughly conducted, some limitations have to be taken into consideration with respect to our study. Firstly, only 15 patients could be included and a greater cohort would have been desirable. It should be noted that several published study populations were even smaller (*n* = 5 [24]; *n* = 10 [9, 28]). Secondly, regarding the results in Table 4, concentrations of methadone and EDDP in hair samples did not increase over five days in all 15 patients, as one would have expected. An explanation may lie in the hair analysis, where only half of the strand was used in each case. Since the drugs may not have been uniformly distributed in the hair, this sampling could have led to variations in concentrations. Moreover, the rate of sweating varies considerably between individuals due to different basic physical conditions and physical activities [27] and even between body areas as well as different degrees of skin contact e.g. due to body hair. This could result in a variability of the amount of drug present in the hair being worn by the same person. Thirdly, the concentrations of EDDP in our patches were obviously lower than those previously published by two working groups [9, 27, 28], resulting in higher methadone-to-EDDP ratios. Possible reasons for this were discussed above. Overall, ranges and ratios were in line with previously published results. In general, comparison of results from different laboratories using different methods has to be done carefully, particularly if a possible conversion of methadone to EDDP cannot be excluded in every GCMS-based method (e.g. if this was not checked in the method development). Moreover, it would have been useful to have obtained hair samples from the participants themselves, to compare methadone to EDDP-ratios in serum, sweat, head hair and in hair from the belt pouches. As explained above, patients did not agree to this extensive sampling. However, Fucci et al. showed that in 60% (6/10) of patients, the ratios from hair and sweat were different [9].

The strength of our study is the simultaneous sampling of blood and sweat together with the sweat-contaminated hair samples, giving a comparison of concentrations and ratios of methadone and EDDP. Additionally, this is the first time that the transfer of a drug and its metabolite via sweat into substance-free hair was proven in a structured prospective study. Importantly, we could show that the ratio in sweat and sweat-contaminated hair need not be the same.

In summary, the route of transfer of methadone and EDDP by methadone substitution patients to drug-free hair could be proven. The question of the transfer route by sweat and close contact also arises, however, for all substances (of abuse) [31]. Very little information is available on transfer of THC to hair both through skin contact/sweat and through cannabis smoke, but without cannabis consumption [32]. Further experiments are necessary to enable the correct interpretation of hair analyses results from children living in a drug-rich environment.

## Conclusion

Our study has shown that an external contamination by transfer of methadone and EPPD from sweat into hair matrix is possible, although there was no correlation found between blood concentrations, sweat concentrations and substance concentrations in hair samples. Following this result, the transfer of methadone and EDDP to children’s hair by sweat from direct contact with drug users is now a plausible explanation for the presence of these substances in children’s hair. Detection of the metabolite EDDP does not necessarily prove an oral intake of methadone by the child. Moreover, there is a high inter-individual variability and a relevant overlapping of methadone/EDDP ratio in sweat and in hair. Therefore, it is not possible to draw conclusions as to the source of the methadone in children’s hair. This study demonstrates once more that results of hair analysis of children must be interpreted particularly carefully.
